# Supplementary material for: A Likelihood Approach for Real-Time Calibration of Stochastic Compartmental Epidemic Models
Source: PLoS Comput Biol. 2017 Jan 17;13(1):e1005257. doi: 10.1371/journal.pcbi.1005257 (PMC5240920; doi:10.1371/journal.pcbi.1005257)
Supplement: S1 File — (TAR.GZ) [file pcbi.1005257.s014.tar.gz › HSPH_Online-SI-Revision/EnKF07/n10-mild/n10-mild_EnKF2_table-4.pdf]

1	newly 3 weeks spec	199.	184.28	{10., 264.}	91.6482	0.60295	newly 3 weeks cumul	583.	935.49	{36., 1175.}	110.753	0.39824	newly 5 weeks spec	174.	83.55	{2., 209.}	80.2586	0.913029	newly 5 weeks cumul	973.	1136.98	{58., 1346.}	86.8695	0.432241	newly 54 weeks cumul	2959.	1388.59	{87., 2476.}	79.1899	0.727145
2	newly 3 weeks spec	442.	159.83	{2., 724.}	93.9932	1.38889	newly 3 weeks cumul	1121.	1237.6	{31., 6501.}	135.886	0.732587	newly 5 weeks spec	488.	24.13	{0., 45.}	95.6865	2.33661	newly 5 weeks cumul	2106.	1316.99	{41., 6872.}	105.462	0.893223	newly 59 weeks cumul	3790.	1375.19	{42., 6879.}	90.9987	1.06472
3	newly 3 weeks spec	438.	130.35	{9., 235.}	83.2808	0.978729	newly 3 weeks cumul	1008.	1958.75	{487., 4890.}	112.072	0.276208	newly 5 weeks spec	488.	26.94	{0., 47.}	94.4795	2.45567	newly 5 weeks cumul	1997.	2043.64	{487., 5285.}	67.995	0.321334	newly 62 weeks cumul	4433.	2082.55	{487., 5300.}	61.559	0.509099
4	newly 3 weeks spec	427.	47.86	{0., 83.}	93.3302	1.94556	newly 3 weeks cumul	1020.	1124.33	{58., 1731.}	135.962	0.716819	newly 5 weeks spec	527.	25.74	{0., 48.}	97.8824	2.69428	newly 5 weeks cumul	2089.	1180.69	{58., 1904.}	105.503	0.911313	newly 57 weeks cumul	4089.	1298.62	{58., 1925.}	93.7036	1.08935
5	newly 3 weeks spec	105.	292.69	{2., 932.}	279.286	0.809543	newly 3 weeks cumul	340.	1758.4	{81., 7097.}	470.724	0.557836	newly 5 weeks spec	120.	108.79	{0., 144.}	136.658	1.1671	newly 5 weeks cumul	593.	2050.24	{87., 8258.}	310.755	0.530253	newly 37 weeks cumul	2494.	2192.47	{89., 9183.}	118.183	0.822111
6	newly 3 weeks spec	329.	316.11	{0., 767.}	98.7508	1.20563	newly 3 weeks cumul	816.	3618.91	{767., 8486.}	349.43	0.550186	newly 5 weeks spec	353.	102.37	{0., 305.}	81.4419	1.84427	newly 5 weeks cumul	1522.	3892.41	{792., 10 016.}	171.415	0.38505	newly 63 weeks cumul	3219.	4068.21	{792., 10 179.}	67.0137	0.268546
7	newly 3 weeks spec	337.	142.87	{2., 678.}	97.6113	1.25478	newly 3 weeks cumul	914.	1260.41	{37., 5221.}	149.29	0.645602	newly 5 weeks spec	288.	26.46	{0., 43.}	94.8403	2.1098	newly 5 weeks cumul	1547.	1342.15	{43., 5451.}	116.889	0.765953	newly 59 weeks cumul	3151.	1405.48	{43., 7004.}	92.3256	0.955089
8	newly 3 weeks spec	276.	167.	{9., 276.}	83.2391	0.70835	newly 3 weeks cumul	750.	967.88	{41., 1401.}	97.2053	0.493541	newly 5 weeks spec	242.	77.15	{2., 142.}	89.186	1.20937	newly 5 weeks cumul	1290.	1155.91	{53., 1656.}	83.3465	0.561854	newly 54 weeks cumul	4074.	1365.86	{62., 2211.}	81.5817	0.89894
9	newly 3 weeks spec	372.	115.18	{10., 133.}	87.4731	0.925469	newly 3 weeks cumul	848.	653.01	{45., 696.}	87.8172	0.551393	newly 5 weeks spec	527.	42.55	{1., 68.}	91.9412	1.58757	newly 5 weeks cumul	1906.	763.55	{60., 847.}	85.6689	0.780316	newly 58 weeks cumul	4128.	838.7	{70., 975.}	85.3343	1.02307
10	newly 3 weeks spec	221.	187.4	{2., 103.}	138.923	1.10771	newly 3 weeks cumul	630.	1385.49	{57., 7131.}	205.23	0.554128	newly 5 weeks spec	246.	55.93	{0., 41.}	100.841	1.881	newly 5 weeks cumul	1140.	1544.62	{73., 7214.}	154.116	0.694682	newly 55 weeks cumul	2746.	1621.78	{86., 7214.}	108.9	0.953378
11	newly 3 weeks spec	268.	270.93	{3., 605.}	146.974	1.14451	newly 3 weeks cumul	729.	1038.78	{20., 4297.}	153.956	0.698695	newly 5 weeks spec	221.	92.03	{0., 346.}	105.48	1.78533	newly 5 weeks cumul	1225.	1301.26	{23., 4339.}	132.767	0.822037	newly 60 weeks cumul	2530.	1437.41	{24., 6011.}	101.106	1.01633
12	newly 3 weeks spec	699.	169.08	{15., 399.}	79.8913	0.936255	newly 3 weeks cumul	1437.	2250.	{725., 6095.}	88.3688	0.269229	newly 5 weeks spec	522.	33.01	{0., 85.}	93.6762	2.06611	newly 5 weeks cumul	2666.	2355.74	{771., 6104.}	65.7929	0.359253	newly 62 weeks cumul	3704.	2386.69	{771., 6104.}	60.5807	0.422394
13	newly 3 weeks spec	431.	238.96	{4., 351.}	93.1926	0.930921	newly 3 weeks cumul	1134.	1158.97	{42., 1322.}	97.8721	0.589243	newly 5 weeks spec	505.	102.56	{1., 213.}	89.4535	1.45162	newly 5 weeks cumul	2205.	1413.76	{49., 1925.}	89.5129	0.742019	newly 54 weeks cumul	4115.	1694.24	{56., 4073.}	84.5239	0.901919
14	newly 3 weeks spec	356.	201.66	{0., 252.}	107.292	1.4088	newly 3 weeks cumul	939.	1262.23	{20., 1956.}	136.879	0.737063	newly 5 weeks spec	351.	72.84	{0., 107.}	92.5926	1.66814	newly 5 weeks cumul	1659.	1455.06	{24., 2080.}	110.316	0.829743	newly 55 weeks cumul	3922.	1573.77	{35., 2253.}	92.8121	1.07484
15	newly 3 weeks spec	198.	69.49	{0., 78.}	95.2475	1.40606	newly 3 weeks cumul	512.	960.	{30., 1891.}	190.098	0.637411	newly 5 weeks spec	238.	47.06	{0., 55.}	99.9832	2.11137	newly 5 weeks cumul	1009.	1060.12	{30., 1891.}	129.913	0.752611	newly 57 weeks cumul	4011.	1379.78	{30., 3647.}	93.7228	1.13048
16	newly 3 weeks spec	383.	96.45	{10., 133.}	86.5509	0.941448	newly 3 weeks cumul	938.	509.43	{46., 693.}	70.7665	0.552277	newly 5 weeks spec	435.	42.19	{2., 76.}	91.2391	1.55548	newly 5 weeks cumul	1811.	611.79	{61., 917.}	79.1657	0.750677	newly 58 weeks cumul	3950.	726.79	{75., 1053.}	85.0605	1.01709
17	newly 3 weeks spec	66.	317.89	{2., 759.}	424.591	0.664029	newly 3 weeks cumul	166.	1371.19	{7., 5199.}	759.139	0.767701	newly 5 weeks spec	111.	143.25	{1., 407.}	138.261	0.800662	newly 5 weeks cumul	390.	1723.94	{12., 6716.}	378.944	0.606938	newly 55 weeks cumul	3319.	2038.03	{15., 6888.}	97.6189	0.943175
18	newly 3 weeks spec	196.	247.61	{0., 307.}	168.413	1.7585	newly 3 weeks cumul	553.	1520.57	{17., 5551.}	235.278	0.625922	newly 5 weeks spec	192.	109.61	{0., 341.}	118.891	1.906	newly 5 weeks cumul	970.	1797.02	{21., 6749.}	163.334	0.626032	newly 55 weeks cumul	2464.	2000.53	{26., 6812.}	107.68	0.835532
19	newly 3 weeks spec	378.	106.68	{4., 330.}	84.5185	1.02616	newly 3 weeks cumul	1189.	594.76	{32., 1079.}	83.2145	0.739067	newly 5 weeks spec	370.	45.64	{2., 74.}	91.6162	1.51432	newly 5 weeks cumul	1992.	709.11	{39., 1649.}	82.9794	0.865287	newly 58 weeks cumul	3457.	805.27	{44., 2613.}	81.8985	1.01135
20	newly 3 weeks spec	289.	139.42	{2., 708.}	101.426	1.17099	newly 3 weeks cumul	900.	1250.45	{32., 5268.}	152.121	0.668949	newly 5 weeks spec	356.	25.9	{0., 39.}	94.9213	2.28413	newly 5 weeks cumul	1637.	1327.17	{46., 5268.}	115.06	0.811441	newly 59 weeks cumul	3969.	1391.72	{53., 6684.}	88.2736	1.05612
21	newly 3 weeks spec	420.	164.81	{2., 704.}	98.6595	1.33239	newly 3 weeks cumul	1015.	1290.13	{27., 7654.}	151.12	0.735783	newly 5 weeks spec	456.	28.24	{0., 38.}	95.5877	2.36121	newly 5 weeks cumul	1966.	1381.1	{31., 7907.}	114.455	0.913596	newly 59 weeks cumul	3646.	1444.17	{35., 7908.}	96.0036	1.09415
22	newly 3 weeks spec	279.	248.97	{2., 402.}	108.749	0.89948	newly 3 weeks cumul	788.	1270.65	{18., 2000.}	153.131	0.719363	newly 5 weeks spec	256.	90.75	{1., 126.}	88.7148	1.18085	newly 5 weeks cumul	1343.	1509.4	{24., 2332.}	120.807	0.776777	newly 55 weeks cumul	3250.	1668.52	{28., 3097.}	93.6443	0.996281
23	newly 3 weeks spec	399.	143.04	{2., 184.}	108.165	1.50627	newly 3 weeks cumul	1073.	591.96	{14., 1136.}	104.39	0.915707	newly 5 weeks spec	394.	52.25	{0., 79.}	95.1599	2.38825	newly 5 weeks cumul	1876.	740.28	{16., 1576.}	99.2942	1.08839	newly 60 weeks cumul	3098.	812.8	{17., 1844.}	93.8644	1.25736
24	newly 3 weeks spec	199.	184.2	{3., 483.}	109.196	0.846258	newly 3 weeks cumul	621.	774.67	{41., 1318.}	97.2415	0.472867	newly 5 weeks spec	188.	105.89	{0., 233.}	105.739	1.51176	newly 5 weeks cumul	1038.	1024.2	{43., 2210.}	95.0501	0.583578	newly 56 weeks cumul	2085.	1328.89	{43., 5377.}	92.576	0.790098
25	newly 3 weeks spec	253.	261.23	{0., 743.}	104.968	1.23993	newly 3 weeks cumul	550.	3898.04	{757., 10 640.}	613.316	0.705755	newly 5 weeks spec	366.	75.48	{0., 276.}	84.3934	1.95487	newly 5 weeks cumul	1317.	4104.18	{788., 10 640.}	227.86	0.441091	newly 63 weeks cumul	2982.	4265.89	{788., 11 094.}	84.6026	0.309584
26	newly 3 weeks spec	272.	182.81	{39., 222.}	90.739	0.546996	newly 3 weeks cumul	780.	676.84	{144., 772.}	82.2359	0.390336	newly 5 weeks spec	259.	104.45	{11., 189.}	88.9768	0.829678	newly 5 weeks cumul	1322.	922.67	{202., 1143.}	84.5499	0.485492	newly 54 weeks cumul	2407.	1326.64	{325., 2686.}	85.1799	0.606592
27	newly 3 weeks spec	426.	155.75	{8., 295.}	92.5282	0.987855	newly 3 weeks cumul	1154.	941.02	{211., 1713.}	105.087	0.60666	newly 5 weeks spec	412.	67.06	{0., 89.}	94.9272	1.67362	newly 5 weeks cumul	2041.	1106.55	{223., 2092.}	94.2151	0.740118	newly 56 weeks cumul	3536.	1266.	{223., 2285.}	90.927	0.911061
28	newly 3 weeks spec	120.	209.72	{1., 700.}	172.55	0.813744	newly 3 weeks cumul	339.	810.84	{71., 2894.}	185.127	0.413587	newly 5 weeks spec	111.	128.78	{0., 253.}	136.27	1.28262	newly 5 weeks cumul	576.	1106.25	{71., 3778.}	162.783	0.488012	newly 34 weeks cumul	2091.	1542.79	{71., 4550.}	94.2319	0.732969
29	newly 3 weeks spec	361.	58.64	{0., 84.}	94.2161	1.77194	newly 3 weeks cumul	1007.	1201.52	{74., 2527.}	143.754	0.74277	newly 5 weeks spec	360.	38.92	{0., 57.}	99.0389	2.48423	newly 5 weeks cumul	1758.	1284.84	{78., 2671.}	115.196	0.871478	newly 57 weeks cumul	3678.	1460.04	{78., 2671.}	96.3045	1.04497
30	newly 3 weeks spec	367.	166.09	{0., 153.}	103.872	1.33442	newly 3 weeks cumul	970.	1088.07	{213., 1342.}	119.832	0.495981	newly 5 weeks spec	361.	66.61	{0., 52.}	101.105	1.86795	newly 5 weeks cumul	1701.	1267.5	{228., 1506.}	107.406	0.656068	newly 56 weeks cumul	3445.	1345.68	{228., 1567.}	95.8502	0.892828
31	newly 3 weeks spec	288.	343.33	{0., 815.}	127.733	1.32181	newly 3 weeks cumul	670.	4311.66	{764., 9427.}	544.349	0.665407	newly 5 weeks spec	360.	108.83	{0., 331.}	87.1639	2.00036	newly 5 weeks cumul	1418.	4602.34	{795., 10 728.}	238.874	0.451123	newly 63 weeks cumul	2824.	4818.81	{796., 11 739.}	101.489	0.30603
32	newly 3 weeks spec	126.	91.83	{4., 45.}	129.69	0.967339	newly 3 weeks cumul	395.	630.91	{203., 421.}	114.296	0.254321	newly 5 weeks spec	145.	18.96	{0., 11.}	98.9379	2.33452	newly 5 weeks cumul	687.	693.39	{203., 452.}	107.323	0.447079	newly 36 weeks cumul	3008.	709.8	{203., 454.}	91.8531	0.995596
33	newly 3 weeks spec	466.	50.77	{0., 80.}	94.2639	1.92719	newly 3 weeks cumul	1168.	1341.11	{70., 4381.}	145.482	0.773887	newly 5 weeks spec	533.	26.88	{0., 47.}	98.0375	2.74124	newly 5 weeks cumul	2236.	1399.36	{70., 4586.}	110.502	0.926645	newly 57 weeks cumul	4145.	1512.34	{70., 4586.}	93.2338	1.05295
34	newly 3 weeks spec	301.	88.57	{0., 91.}	98.0565	1.57527	newly 3 weeks cumul	838.	1378.79	{59., 3549.}	174.734	0.656436	newly 5 weeks spec	335.	56.14	{0., 56.}	101.152	2.3161	newly 5 weeks cumul	1525.	1498.73	{59., 4732.}	130.986	0.788273	newly 57 weeks cumul	3835.	1725.55	{59., 5002.}	97.0495	0.99802
35	newly 3 weeks spec	430.	89.01	{0., 91.}	94.486	1.69383	newly 3 weeks cumul	1171.	1097.28	{86., 1915.}	125.085	0.780156	newly 5 weeks spec	358.	50.4	{0., 54.}	97.933	2.20991	newly 5 weeks cumul	1960.	1207.11	{87., 2893.}	105.355	0.883362	newly 57 weeks cumul	3634.	1464.01	{87., 4064.}	94.8539	1.00967
36	newly 3 weeks spec	452.	124.84	{2., 517.}	91.0398	1.30643	newly 3 weeks cumul	1197.	1015.32	{133., 3599.}	112.045	0.704934	newly 5 weeks spec	398.	26.86	{0., 35.}	95.0754	2.28311	newly 5 weeks cumul	2080.	1092.75	{147., 3602.}	95.6793	0.844443	newly 59 weeks cumul	3700.	1198.44	{150., 5252.}	87.3508	1.01021
37	newly 3 weeks spec	333.	144.01	{7., 174.}	91.5766	0.879807	newly 3 weeks cumul	782.	1946.64	{498., 4613.}	163.148	0.346992	newly 5 weeks spec	357.	35.92	{0., 30.}	96.0896	2.34959	newly 5 weeks cumul	1618.	2048.52	{499., 4615.}	78.9419	0.297331	newly 62 weeks cumul	2716.	2091.85	{499., 4615.}	67.9643	0.405773
38	newly 3 weeks spec	369.	98.19	{6., 131.}	84.7073	0.95608	newly 3 weeks cumul	949.	516.15	{36., 717.}	71.1117	0.597726	newly 5 weeks spec	348.	41.82	{1., 78.}	90.2816	1.45992	newly 5 weeks cumul	1684.	621.6	{52., 977.}	77.5475	0.75757	newly 58 weeks cumul	4183.	731.76	{62., 1228.}	84.7263	1.073
39	newly 3 weeks spec	526.	137.54	{3., 742.}	92.4335	1.39444	newly 3 weeks cumul	1168.	1008.19	{167., 2836.}	111.574	0.65237	newly 5 weeks spec	460.	44.88	{0., 42.}	96.3043	2.36667	newly 5 weeks cumul	2156.	1127.2	{182., 3717.}	95.9824	0.812183	newly 59 weeks cumul	3091.	1245.48	{225., 3956.}	91.3782	0.909914
40	newly 3 weeks spec	95.	141.66	{6., 407.}	153.453	0.653753	newly 3 weeks cumul	322.	2203.21	{84., 9021.}	624.09	0.68358	newly 5 weeks spec	79.	6.12	{0., 15.}	93.3418	1.81774	newly 5 weeks cumul	527.	2240.48	{87., 9265.}	377.066	0.622415	newly 32 weeks cumul	1882.	2242.26	{89., 9307.}	116.366	0.636764
41	newly 3 weeks spec	565.	310.58	{0., 759.}	82.7965	1.35387	newly 3 weeks cumul	1036.	3685.82	{726., 9145.}	269.131	0.474007	newly 5 weeks spec	573.	93.87	{0., 299.}	87.918	2.16964	newly 5 weeks cumul	2224.	3942.22	{749., 10 078.}	108.791	0.31967	newly 63 weeks cumul	3132.	4117.81	{751., 10 153.}	77.9824	0.307614
42	newly 3 weeks spec	232.	108.4	{0., 87.}	101.724	1.21349	newly 3 weeks cumul	680.	1266.84	{272., 2585.}	147.968	0.315841	newly 5 weeks spec	278.	16.55	{0., 31.}	94.0612	2.12413	newly 5 weeks cumul	1255.	1325.44	{280., 2600.}	111.753	0.468985	newly 55 weeks cumul	2911.	1338.86	{287., 2600.}	91.0326	0.736208
43	newly 3 weeks spec	534.	23.68	{0., 64.}	95.5655	2.23	newly 3 weeks cumul	1357.	578.72	{127., 1346.}	65.1069	0.562327	newly 5 weeks spec	396.	6.24	{0., 11.}	98.4242	2.78296	newly 5 weeks cumul	2311.	596.01	{143., 1346.}	75.4959	0.754753	newly 61 weeks cumul	3391.	607.44	{146., 1346.}	82.3262	0.909994
44	newly 3 weeks spec	300.	38.28	{0., 80.}	91.3333	1.77603	newly 3 weeks cumul	794.	1104.38	{67., 3189.}	153.539	0.640709	newly 5 weeks spec	309.	12.76	{0., 51.}	95.8706	2.52963	newly 5 weeks cumul	1422.	1136.07	{67., 3191.}	118.222	0.787803	newly 57 weeks cumul	3738.	1196.13	{67., 3191.}	92.5054	1.05269
45	newly 3 weeks spec	534.	569.95	{95., 1070.}	68.7921	0.368982	newly 3 weeks cumul	994.	4018.58	{587., 9332.}	318.783	0.546705	newly 5 weeks spec	717.	150.9	{5., 359.}	79.6039	1.06758	newly 5 weeks cumul	2450.	4447.78	{658., 10 517.}	112.818	0.352524	newly 64 weeks cumul	4278.	4694.22	{794., 10 737.}	63.5049	0.322925
46	newly 3 weeks spec	331.	50.33	{0., 79.}	95.5015	2.04949	newly 3 weeks cumul	825.	794.49	{137., 1534.}	73.0776	0.381717	newly 5 weeks spec	370.	15.92	{0., 13.}	96.973	2.69882	newly 5 weeks cumul	1578.	839.35	{149., 1612.}	71.3188	0.553914	newly 61 weeks cumul	3207.	860.02	{150., 1731.}	78.9454	0.812401
47	newly 3 weeks spec	416.	20.61	{0., 60.}	95.0457	2.08447	newly 3 weeks cumul	828.	650.59	{170., 1323.}	54.3853	0.329832	newly 5 weeks spec	448.	4.08	{0., 11.}	99.0893	2.91673	newly 5 weeks cumul	1770.	663.64	{175., 1323.}	67.4124	0.571591	newly 61 weeks cumul	4274.	671.52	{183., 1323.}	84.2883	0.932197
48	newly 3 weeks spec	360.	91.39	{11., 122.}	83.7639	0.918836	newly 3 weeks cumul	945.	570.17	{47., 577.}	77.4085	0.551316	newly 5 weeks spec	310.	32.69	{2., 68.}	90.9839	1.43874	newly 5 weeks cumul	1682.	655.62	{59., 701.}	81.2235	0.715777	newly 58 weeks cumul	3197.	723.83	{69., 829.}	84.335	0.923654
49	newly 3 weeks spec	197.	131.91	{27., 158.}	75.9442	0.435798	newly 3 weeks cumul	473.	542.61	{106., 622.}	68.6829	0.243743	newly 5 weeks spec	183.	71.34	{14., 121.}	83.3989	0.716336	newly 5 weeks cumul	887.	709.01	{156., 834.}	68.5986	0.345229	newly 54 weeks cumul	3344.	1013.87	{225., 1430.}	84.0906	0.771711
50	newly 3 weeks spec	233.	139.41	{7., 148.}	100.536	0.790783	newly 3 weeks cumul	705.	550.28	{38., 613.}	87.1631	0.532374	newly 5 weeks spec	204.	64.98	{0., 73.}	95.0098	1.15426	newly 5 weeks cumul	1157.	711.	{43., 778.}	90.5497	0.643824	newly 54 weeks cumul	2093.	899.94	{64., 1016.}	88.9298	0.784773
